# Gene-Guided Treatment Decision-Making in Non-Small Cell Lung Cancer – A Systematic Review

**DOI:** 10.3389/fonc.2021.754427

**Published:** 2021-10-12

**Authors:** Jatta Saarenheimo, Heidi Andersen, Natalja Eigeliene, Antti Jekunen

**Affiliations:** ^1^Department of Pathology, Vaasa Central Hospital, Vaasa, Finland; ^2^Department of Oncology, Vaasa Central Hospital, Vaasa, Finland; ^3^Tema Cancer, Karolinska University Hospital, Stockholm, Sweden; ^4^Faculty of Medicine and Health Technology, Tampere University, Tampere, Finland; ^5^Department of Oncology and Radiotherapy, Turku University, Turku, Finland

**Keywords:** shared decision making, non-small cell lung cancer, genes, liquid biopsy, targeted therapy

## Abstract

Decision-making in cancer treatment is part of clinicians’ everyday work, and it is especially challenging in non-small cell lung cancer (NSCLC) patients, for whom decisions are clearly dependent on gene alterations or the lack of them. The multimodality of treatments, involvement of gene alterations in defining systemic cancer therapies, and heterogeneous nature of tumors and their responsiveness provide extra challenges. This article reviews the existing literature to 2021 with extra effort to explore the role of genes and gene-driven therapies as part of decision-making. The process and elements in this decision-making participation are recognized and discussed comprehensively. Genetic health literacy aids are provided as a part of the review. Our systematic review, data extraction and analysis found that with current methods and broad gene panels, patients benefit from early molecular testing of liquid biopsy samples. An estimated 79% of liquid biopsy samples showed somatic mutations based on 8 original studies included in the systematic review. When both liquid biopsy samples and tissue samples are evaluated, the sensitivity to detect targetable mutations in NSCLC increases. We recommend early testing with liquid biopsy. Additional effort is needed for the logistics of obtaining and evaluating samples, and tissue samples should be saved and stored for tests that are not possible from liquid biopsy.

## Introduction

Making medical treatment decisions is one of the key elements in clinicians’ work. Nearly everything that a physician does serves this purpose. Precise cancer diagnostics, with staging, histology, genetic profiling of the tumor and the promise of personalized genomic medicine to guide treatment choice, together with knowledge of a person’s genes and their activity, are becoming clinical standards. Even when treatment is already being administered, there needs to be constant evaluation regarding whether to continue, pause, or even change the ongoing treatment based on side effects, the general condition of a patient and treatment response evaluations. In both cases, the above new genetic tests can improve our decisions.

Based on patient values and life situations, optimal evidence-based treatment recommendations can be offered and discussed with a patient. The patient is an active participant in diagnostic, treatment and surveillance stages. Which treatment to choose from multiple choices based on the harm/benefit ratio needs to be worked through with the patients and their family members. Gene-guided treatment decisions require health literacy, and numeracy and education are needed due to the large amount of information.

Informed decision-making is a process to help patients understand diseases and their associated treatment benefits, risks, limitations, alternatives and uncertainties. Diagnostic tests and treatments need to make sense emotionally and practically for the patient. The patient’s context, values, and preferences need to be considered in shared decision-making. Health literature on genomic terms, such as polygenetic risk profiling, might be lacking. Structural data collection and artificial intelligence with pattern recognition are underused in clinical practice.

This systematic narrative review provides an overview of recent literature about gene-guided treatment decision-making from a respiratory oncologist’s perspective. The aim of the review is to structure existing knowledge and to evaluate gaps in the literature in gene-guided non-small-cell lung cancer diagnostics. The discussion will expand the findings to reflect attitudes in the clinical practice of oncology. The aim of the review was to answer the following population, intervention, comparator, outcome (PICO) research question: Do non-small-cell lung cancer patients benefit from genetic testing with early liquid biopsy, only tissue biopsies or both in terms of treatment decision-making?

## Methods

**Figure 1:**
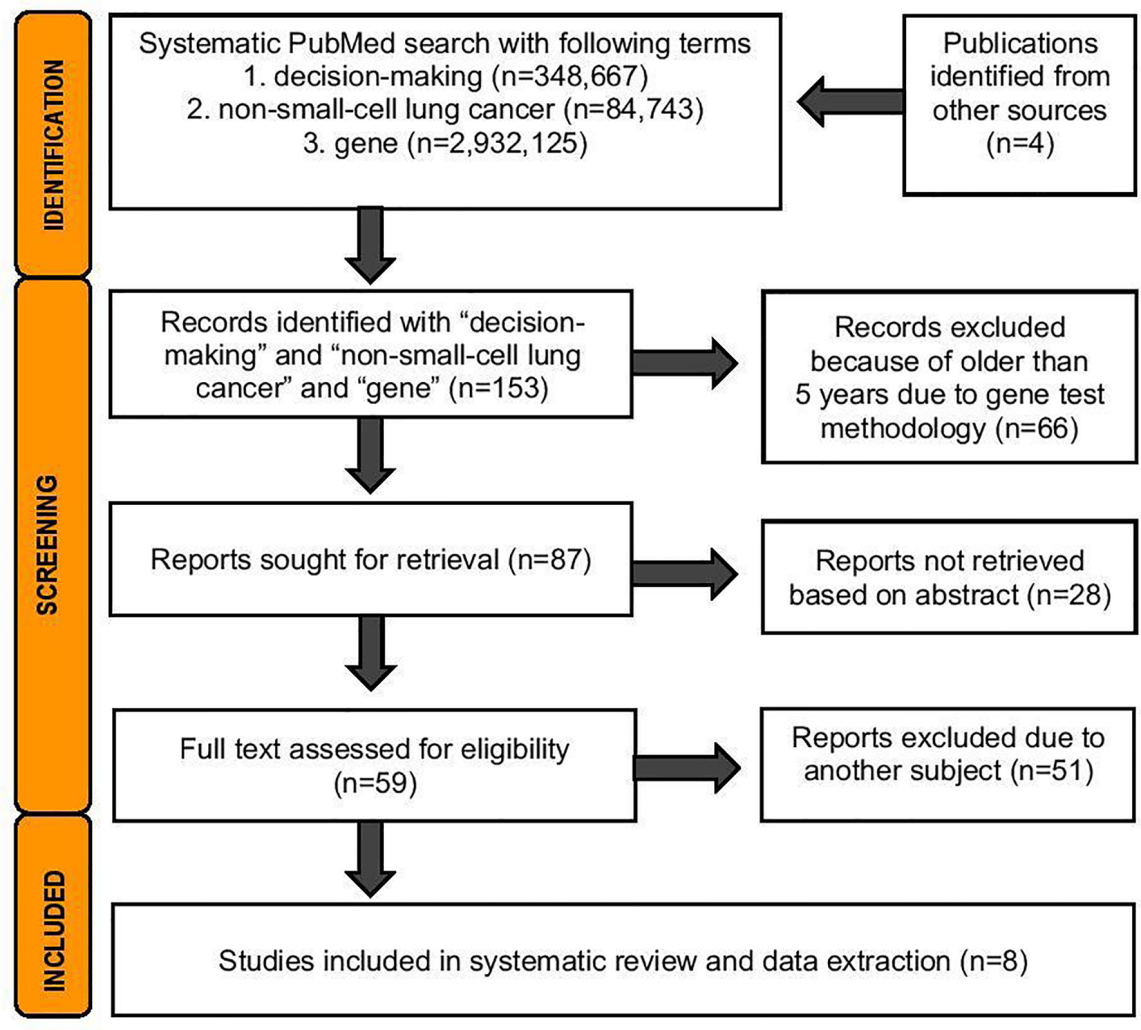
The search for this review was conducted according to the Prisma Statement Checklist.

**Figure 2:**
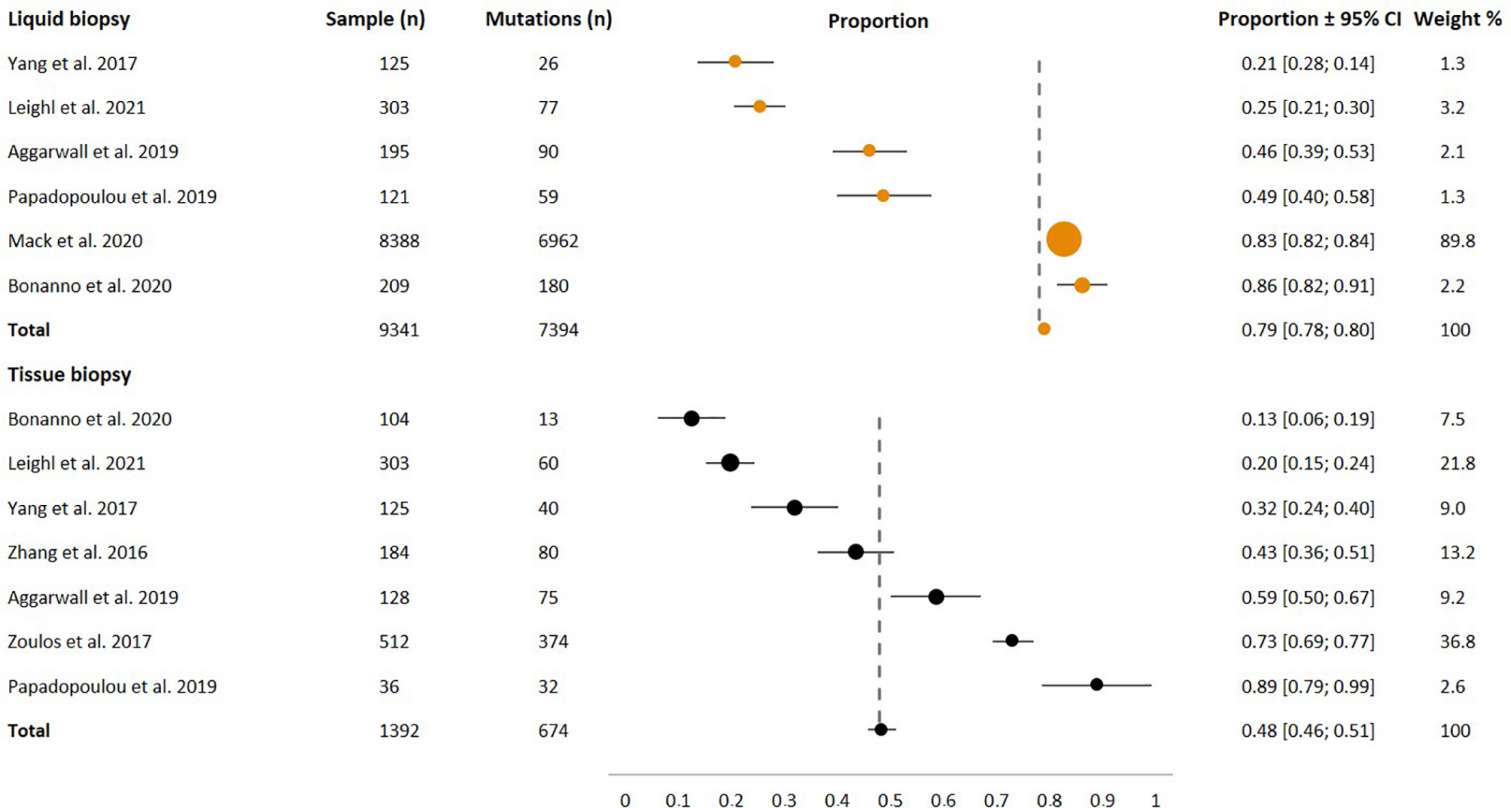
Forest plot of the extracted data from 8 studies: 6 studies utilizing liquid biopsy samples (n = 9341), and 7 studies utilizing tissue biopsy samples (n = 1392). Dashed line shows the weighted mean proportion of somatic mutations (0.79 for liquid biopsy and 0.48 for tissue biopsy).

A systematic literature search was performed 19.5.2021 from PubMed database. The search was conducted with the terms *medical decision* and *non-small-lung cancer* and *gene* according the Prisma Statement Checklist (**Figure 1**). For this analysis, exclusion criteria were publications in languages other than English and trials involving nonhuman subjects. The search was done to cover publications from the last 5 years to be included in the review, due to technical improvements in genetic analyses. Two independent reviewers (JS and HA) analyzed the abstracts of 87 publications and the full texts of 59 publications. The inclusion criteria were that the study had to contain sequencing data from lung cancer patients targeting somatic mutations, either from tissue biopsy or liquid biopsy or from both sample types. Based on relevant information, eight original studies were included in further narrative analyses and data extraction (**Figure 2** and **Supplementary Table 1**).

Extracted variables included the following: publication year, authors, used method, number of patients and number of reported mutations (**Supplementary Table 1**). The proportion of somatic mutations with 95% confidence interval (95% CI) was calculated for each study separately, and presented in Forest plot (**Figure 2**). In addition, the weighted proportion was assessed for both sample types.

## Results

### Medical Decision-Making Clinical Cancer Gene

A search performed on 19 May 2021 of the term gene on PubMed produced more than two million hits, and the addition of the term non-small-cell lung cancer produced almost one hundred thousand hits. With decision-making and non-small-cell lung cancer, there were 1055 hits, a number that then decreased dramatically when the search for terms included gene (**Figure 1**). We had no hits if we included shared in the search.

### NSCLC Screening

Cancer can be detected from blood with several methods, such as DNA sequencing and methylation, protein, and fragmented DNA analyses. These techniques have limited data on their usefulness in cancer screening. DNA evaluation of fragments for early interception (DELFI) is a noninvasive screening method in which cell-free DNA is isolated from plasma, and minute changes can be detected from these cell-free DNA fragments with genome-wide sequencing. With advanced machine-learning methods, these changes can distinguish cancer and different cancer types (1). The first prospective preliminary results in a lung cancer cohort are presented in the ASCO 2021 abstract. Combining DELFI as a prescreen for low-dose CT increased specificity from 58% to 80% for CT alone vs the combined approach. Additionally, these genome-wide fragmentation profiles can be used to distinguish small-cell lung cancer from non-small-cell lung cancer with high accuracy. Methylation and epigenetic alterations are crucial to oncogenesis and the regulation of gene expression in non-small-cell lung carcinoma (NSCLC). These methylation/expression data can potentially be used as biomarkers to provide molecular-level prediction of lung cancer combined with other risk factors (2, 3).

### NSCLC Diagnostics

More extensive tumor sequencing can identify more mutations. The presence of actionable genetic alterations and treatable mutations can improve clinical decision-making in routine cancer care. Multigene next-generation sequencing (NGS)-targeted panels should be preferred to standard diagnostic techniques for cell-free DNA (cfDNA) profiling in patients who lack tumor tissue for genotyping because they allow the identification of many actionable genetic alterations in a single analysis. NGS methodologies can also reveal the presence of complex genomic variants in cfDNA, such as gene fusions and rearrangements; however, technological advances are mandatory to improve copy number variation (CNV) detection. The genomic profile of NSCLC patients obtained with NGS testing of plasma is similar to that obtained with tissue testing, and even more importantly, plasma NGS assay integration into routine management increases the detection of clinically relevant mutations (3).

### Overview of Studies 1-8

We included eight studies for systemic review and analyses of the diagnostic capacity of liquid biopsy compared to that of tissue biopsy when identifying mutations and treatable mutations. These studies are highly heterogenic, and tissue biopsies have a problem in sample inclusion. Almost half of tissue biopsies do not qualify for further genetic analysis after primary diagnosis due to insufficient samples and problems in tissue handling. In the comparison, the diagnostic value of tissue biopsies is overestimated. Overall, studies have shown that more or the same frequency somatic mutations are found in patient samples. However, it is notable that the matched samples from one patient are not always concordant (Study 6). The analysis of sensitivity plays a crucial role, and the results from earlier studies need to be evaluated when observing the results. Both liquid biopsy samples and tissue biopsy samples might result in false-negative results. The diagnostic accuracy and sensitivity seem to show an increasing trend with time, and the broadest panel available should be used in primary diagnoses (**Figure 2** and **Supplementary Table 1**). Thus, overall, the studies showed that NGS panels could be used for tumor molecular profiling, and the results were further applied in personalized treatment decision-making for NSCLC patients (**Figure 2** and **Supplementary Table 1**). When analyzed in detail, these studies have different viewpoints.

Study 1 described that by using a target assay, it is possible to diagnose treatable mutations from tissue samples (4). Study 2 described positive and negative predictive values for epidermal growth factor receptor (*EGFR)* mutations in cfDNA of 91.2% (31/34) and 67.1% (49/73) and for B-Raf proto-oncogene serine/threonine kinase (*BRAF)* mutations of 22.2% (2/9) and 94.9% (93/98), respectively (5). Study 3 showed that 51.6% of the patients had a mutation in a gene that could be related to off-label therapy or an indication for access to a clinical trial (6). Additionally, in study 4, the integration of plasma NGS testing into the routine management of stage IV NSCLC demonstrated a marked increase in the detection of therapeutically targetable mutations and improved delivery of molecular-guided therapy (7). Study 5 indicated the feasibility of circulating tumor DNA (ctDNA) analysis as a tumor biopsy surrogate in clinical practice for NSCLC personalized treatment decision-making, with a high positive predictive value of 88.9% (8). These analyses were targeted, and ctDNA was measured instead of cfDNA. Study 6 visualized the problems clinicians are facing daily. Out of 303 patients, NGS was performed on plasma samples in 209 patients; 78 of these were also evaluated in tissue for druggable alterations. Half of the tissue samples were deemed not evaluable. The results of NGS testing affected treatment decisions in 1:4 to 1:5 patients (9).

The largest liquid biopsy study, study 7, showed genetic alterations in 86% of samples, with 57.2% showing more than one mutation. *EGFR* and Kirsten rat sarcoma viral oncogene homolog (*KRAS)* mutations were sometimes detected simultaneously but with mutual exclusivity for *BRAF*, Ret Proto-Oncogene (*RET)*, anaplastic lymphoma kinase (*ALK)*, and c-ros oncogene 1 (*ROS1)*. The testing of cfDNA increased the detection rate of treatable genomic alterations by 65% in the study in patients for whom both tissue and cfDNA testing results were known; however, the actual data on tissue samples were deficient. The authors speculated that the increase was mainly due to undergenotyping due to insufficient tissue (10). Last, study 8 reported that when using guideline-driven cfDNA analysis in addition to tumor tissue testing, the detection of treatable genomic alterations increased by 48% (11).

In addition to NGS panels, immunohistochemistry (IHC) of programmed death-ligand 1 (PD-L1) expression is recommended for the evaluation of immunotherapy treatment. The test has limitations as a predictive marker for treatment responsiveness (12). For example, the PD-L1 thresholds were variable both within and across tumor types using several different assays, and PD-L1 expression was also measured in a variable fashion in either tumor cells or tumor-infiltrating immune cells (13). Therefore, it is suggested that other biomarkers, such as tumor mutation burden and neoantigens, as well as biomarkers reflecting the host environment and microbiome or tumor inflamed microenvironment, including gene expression signatures, should be explored as more reliable and accurate alternatives to PD-L1 IHC for guiding treatment selection (12).

## Discussion

Early liquid biopsy is beneficial in NSCLC, and when combined with genetic testing, diagnostic accuracy increases. In the last five years, development was observed from testing for individual driver mutations toward multigene profiling with panels including 70 gene-guiding treatment choices. In 2021, it can be concluded that gene profiling data should be included in clinical practice guidelines, and enormous research activity is ongoing worldwide, as technical development has given researchers the opportunity to analyze genes in a nonexpensive way and faster than ever. As the information expands, we need structural tools to collect the data and artificial intelligence to analyze the data. Humans are needed for empathic treatment decisions, as medicine is quite seldom an exact science.

### Importance of Guidelines

Criticism has arisen about widely used clinical practice guidelines (CPGs), especially regarding the quality of guidelines. In oncology, CPGs have had a measurable positive effect on clinical practice and outcomes (14), and the demand for more guidelines on a broader range of topics continues to increase (15, 16). The hierarchy of the evidence and the harm/benefit ratio should be considered in evidence-based gene-driven medicine. Guidelines and treatment algorithms are useful tools when determining what is the best for one certain patient (17, 18). Specifically, in lung cancer treatment, it has been shown that by following restrictive guidelines and not giving treatments without predetermined rules, remarkable savings can be achieved (19). By following guidelines, it is possible to improve lung cancer surgery results (20). Based on the literature, the guidelines should incorporate liquid biopsy early in the diagnostic process, with a broad panel, and the local logistics should be improved for better quality of the samples. Genetic testing techniques improve rapidly, and they are difficulties in updating guidelines. Genetic testing is needed for inclusion in clinical trials. For health equity, these tests should be available for all patients with low cost, and the cost should be covered with insurance.

### Physician’s Role in Decision-Making

The oncological treatment process has many patient contact situations. These situations, including diagnostic processes, treatment initiations, and control visits, make it possible to clarify and explain planned tests and treatments to patients and to increase health literacy and numeracy in genetic testing. Often, data increase during the diagnostic pathway, and the stage of disease changes with treatments and time. With expanding treatment options, there is a need for a new approach that includes both genetic information and patient values.

Overall, treatment decisions tend to be subjective, and many factors influence the individual thinking process. There is a tension between wanting to provide each individual patient with the best possible and most appropriate treatment options and the lack of “proof” on which to base such judgments about what is truly the “best possible” or even “appropriate” (21). The knowledge and skills physicians acquired in medical school need to be updated (22). An understanding of new terms in current gene-targeted treatments and diagnostics is needed (**Table 1**). Many factors affect treatment decisions. These factors include that people have different risks of morbidity or mortality due to their genomic profiles, different targetable cancer gene mutations, and comorbidities, together with socioeconomic and behavioral factors that affect the choice of treatment.

**Table 1:** Explanations of common genetic terms used in patient education.

**Actionable genetic alterations**	Potentially responsive to a targeted therapy
**CNVs**	Copy number variation is a phenomenon of a structural variation in a genome, such as a duplication and deletion
**cfDNA**	Cell-free DNA are DNA fragments found in plasma
**ctDNA**	Circulating tumor DNA are DNA fragments originating from a tumor; part of cfDNA
**DELFI**	DNA evaluation of fragments for early interception is a noninvasive cancer screening, where DELFI generates a score that reflects the presence of tumor-derived DNA in plasma based on a multifeature genomic analysis that assesses millions of cfDNA fragments for tumor-derived genomic and epigenomic changes in plasma *via* inexpensive, low-coverage (1-2x) whole-genome sequencing. The test can accurately detect cancer and determine where in the body the tumor has grown through the application of advanced machine-learning methods
**Epigenetics**	The study of how behavior and the environment can cause changes that affect the way genes work
**Genetic characterization**	Genetic characterization detects variations in either DNA sequences or specific genes or modifying factors
**Liquid biopsy**	A liquid biopsy is an assay performed on a sample of body fluid
**NGS**	Next-generation sequencing is a technique to sequence entire genomes or constrained to specific areas of interest
**Methylation**	In methylation, methyl groups are added to the DNA molecule. These methyl groups can change the activity of a DNA segment without changing the sequence
**Oncogene addiction**	A process in which cancers with genetic, epigenetic, or chromosomal irregularities become dependent on one or several genes for maintenance and survival. As a result, cancer cells rely on continuous signaling from these oncogenes for their survival
**Tumor mutation burden (TMB)**	The approximate amount of gene mutation that occurs in the genome of a cancer cell

### Patient’s Impact on Decisions

In modern medicine, patients participate in treatment decisions; thus, communication with patients is essentially important. Finally, it is patients who decide whether a treatment option is initiated based on the information they are given by a doctor. Final decisions rely on patients. Patients need discussions to help them understand medical issues regarding their conditions and treatment recommendations. Discussion should cover treatment options, outcomes, and major prognostic aspects. It is helpful to include an option of re-evaluation in the process, which acknowledges patients’ decisions and any associated uncertainties and is an agreement to re-evaluate the decisions to determine whether and how the selected treatment is working for the patient (23). Genetic health literacy and numeracy require constant education for clinical physicians and for patients to diminish the knowledge gap. With common language, communication is more on point (**Table 1**). Shared decision-making is not just choosing and selecting what to do; it solves problems, and with detailed discussions in a caring way, it is possible to understand what matters to the patient. Justice in health care is achieved by reducing the structural barrier for precision medicine.

## Conclusion

Every doctor would like to treat patients in the best possible way and to have all important treatment options available, and this is not possible without genetic profiling for targeted therapies. Medical information is increasing exponentially, and accessibility to new research has improved dramatically with open science. However, a single doctor’s equipment improves slowly, and the implementation of these tests in diagnostic algorithms and guidelines is needed. As the data available increases, objective decisions would benefit from artificial intelligence. Overall, the trend is moving toward larger genetic testing at an early stage, of both liquid biopsy and tissue biopsy samples, to help and guide personalized treatment decisions and treatment options at different stages of NSCLC.

## Data Availability Statement

The original contributions presented in the study are included in the article/**Supplementary Material**. Further inquiries can be directed to the corresponding author.

## Author Contributions

JS and HA did the systematic reviewing process. All authors contributed to the writing and discussion of the results.

## Funding

This work was supported by the Finnish Governmental Research Fund (EVO).

## Conflict of Interest

The authors declare that the research was conducted in the absence of any commercial or financial relationships that could be construed as a potential conflict of interest.

## Publisher’s Note

All claims expressed in this article are solely those of the authors and do not necessarily represent those of their affiliated organizations, or those of the publisher, the editors and the reviewers. Any product that may be evaluated in this article, or claim that may be made by its manufacturer, is not guaranteed or endorsed by the publisher.
