# Supplementary material for: Gene-Guided Treatment Decision-Making in Non-Small Cell Lung Cancer – A Systematic Review
Source: Front Oncol. 2021 Oct 12;11:754427. doi: 10.3389/fonc.2021.754427 (PMC8546351; doi:10.3389/fonc.2021.754427)
Supplement: Supplementary file 1 [file Table_1.docx]

**Gene-guided treatment decision-making in non-small cell lung cancer – a systematic review**

Jatta Saarenheimo^1^*^†^, Heidi Andersen^2,3,4†^, Natalja Eigeliene^2,5^, Antti Jekunen^2,5^

^1^Department of Pathology, Vaasa Central Hospital, Vaasa, Finland.

^2^Department of Oncology, Vaasa Central Hospital, Vaasa, Finland.

^3^Tema Cancer, Karolinska University Hospital, Stockholm, Sweden.

^4^Tampere University, Tampere, Finland.

^5^Turku University, Turku, Finland.

* Corresponding author: Jatta Saarenheimo, PhD, Department of Pathology, Vaasa Central Hospital, Hietalahdenkatu 2-4, 65130 Vaasa, Finland. jatta.saarenheimo@vshp.fi

^†^ Equal contribution & first authorship

**Supplementary Table 1.** Detailed extracted data from 8 studies included in the review

| **Ref.** | **Liquid biopsy** | **Method** | **Sample (n)** | **Mutations (n)** | **Proportion [± 95% CI]** | **Weight %** |
| --- | --- | --- | --- | --- | --- | --- |
| 1 | Zhang et al. 2016 |  | - | - |  |  |
| 2 | Yang et al. 2017 | CastPCR | 125 | 26 | 0.21 [0.28;0.14] | 1.34 |
| 3 | Zoulos et al. 2017 |  | - | - |  |  |
| 4 | Aggarwall et al. 2019 | Guardant360 | 195 | 90 | 0.46 [0.39; 0.53] | 2.09 |
| 5 | Papadopoulou et al. 2019 | NGS/Oncomine (Thermo) | 121 | 59 | 0.49 [0.40; 0.58] | 1.30 |
| 6 | Bonanno et al. 2020 | Guardant360 | 209 | 180* | 0.86 [0.82; 0.91] | 2.24 |
| 7 | Mack et al. 2020 | Guardant360 | 8388 | 6962 | 0.83 [0.82; 0.84] | 89.80 |
| 8 | Leighl et al. 2021 | Guardant360 | 303 | 77* | 0.25 [0.21; 0.30] | 3.24 |
|  | **Total** |  | 9341 | 7394 | 0.79 [0.78; 0.80] | 100 |
|  |  |  |  | **Weighted proportion** | **0.79** |  |
|  | **Tissue biopsy** |  |  |  |  |  |
| 1 | Zhang et al. 2016 | SMART assay | 184 | 80 | 0.43 [0.36; 0.51] | 13.22 |
| 2 | Yang et al. 2017 | CastPCR | 125 | 40 | 0.32 [0.24; 0.40] | 8.98 |
| 3 | Zoulos et al. 2017 | IonAmpliSeq | 512 | 374 | 0.73 [0.69; 0.77] | 36.78 |
| 4 | Aggarwall et al. 2019 | NGS | 128 | 75 | 0.59 [0.50; 0.67] | 9.20 |
| 5 | Papadopoulou et al. 2019 | NGS/Ion Ampliseq (Thermo) | 36 | 32 | 0.89 [0.79; 0.99] | 2.59 |
| 6 | Bonanno et al. 2020 | Oncomine (Focus assay, 59 gene) | 104 | 13* | 0.13 [0.06; 0.19] | 7.47 |
| 7 | Mack et al. 2020 |  | - | - |  |  |
| 8 | Leighl et al. 2021 | SOC tissue genotyping | 303 | 60* | 0.20 [0.15; 0.24] | 21.77 |
|  | **Total** |  | 1392 | 674 | 0.48 [0.46; 0.51] | 100 |
|  |  |  |  | **Weighted proportion** | **0.48** |  |

*only treatable mutations included
